# Aggressive squamous cell carcinoma of lip in a village woman

**DOI:** 10.11604/pamj.2021.39.224.30137

**Published:** 2021-08-02

**Authors:** Rohan Kumar Singh, Gaurav Vedprakash Mishra

**Affiliations:** 1 Department of Radiodiagnosis, Jawaharlal Nehru Medical College, Datta Meghe Institute of Medical Sciences, Sawangi (Meghe), Wardha, India

**Keywords:** Squamous cell carcinoma, lip, aggressive

## Image in medicine

A 33-year-old female visited the outpatient department of Acharya Vinoba Bhave Rural Hospital (AVBRH), Sawangi with chief complains of painful ulcer over lower lip. On taking proper history the patient said she was apparently alright 4 months back when she noticed a painful non healing ulcer over lower the front region of jaw which was initially small in size and gradually increased to the present size of 5.3 x 2.3cm approximately. Patient had history of pain which was gradual in onset, dull aching, intermittent and localized in nature. Pain aggravated on mastication and relieved after taking medications. Patient also had history of change in quantity and consistency of saliva from normal to thick and ropy with intermittent bleed and pus discharge since 3months. On clinical examination face was asymmetrical due to soft tissue defect present over lower lip with irregular everted margins. On incisional biopsy of lesion done under local anaesthesia on 15/09/20 revealed squamous cell carcinoma of lower lip.

**Figure 1:**
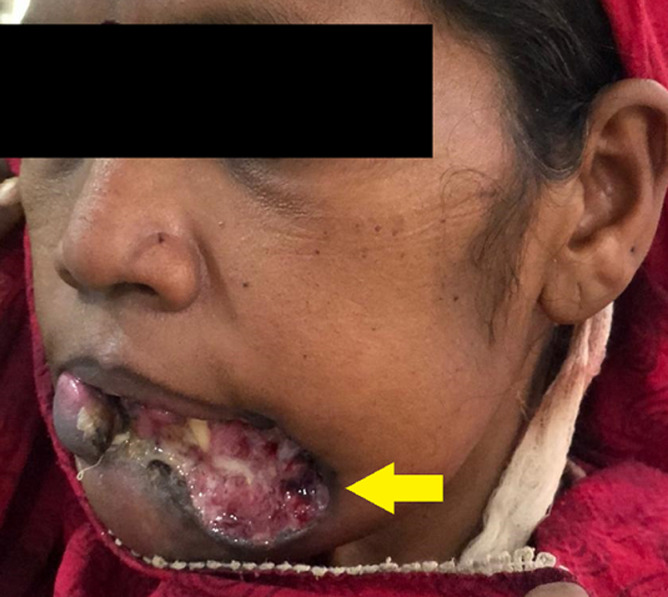
asymmetrical soft tissue defect present over lower lip with irregular everted margins and ulcerative lesion-aggressive Squamous cell carcinoma of lower lip

